# Information-Driven Gas Distribution Mapping for Autonomous Mobile Robots

**DOI:** 10.3390/s23125387

**Published:** 2023-06-07

**Authors:** Andres Gongora, Javier Monroy, Faezeh Rahbar, Chiara Ercolani, Javier Gonzalez-Jimenez, Alcherio Martinoli

**Affiliations:** 1 Machine Perception and Intelligent Robotics (MAPIR) Research Group, Malaga Institute for Mechatronics Engineering and Cyber-Physical Systems (IMECH.UMA), University of Malaga, 29071 Malaga, Spain; andresgongora@uma.es (A.G.); javiergonzalez@uma.es (J.G.-J.); 2 Distributed Intelligent Systems and Algorithms Laboratory (DISAL), School of Architecture, Civil and Environmental Engineering, École Polytechnique Fédérale de Lausanne, 1015 Lausanne, Switzerland; faezeh.rahbar@gmail.com (F.R.); chiara.ercolani@epfl.ch (C.E.); alcherio.martinoli@epfl.ch (A.M.)

**Keywords:** gas distribution mapping, mobile robot olfaction, estimation theory, environmental monitoring

## Abstract

The ability to sense airborne pollutants with mobile robots provides a valuable asset for domains such as industrial safety and environmental monitoring. Oftentimes, this involves detecting how certain gases are spread out in the environment, commonly referred to as a gas distribution map, to subsequently take actions that depend on the collected information. Since the majority of gas transducers require physical contact with the analyte to sense it, the generation of such a map usually involves slow and laborious data collection from all key locations. In this regard, this paper proposes an efficient exploration algorithm for 2D gas distribution mapping with an autonomous mobile robot. Our proposal combines a Gaussian Markov random field estimator based on gas and wind flow measurements, devised for very sparse sample sizes and indoor environments, with a partially observable Markov decision process to close the robot’s control loop. The advantage of this approach is that the gas map is not only continuously updated, but can also be leveraged to choose the next location based on how much information it provides. The exploration consequently adapts to how the gas is distributed during run time, leading to an efficient sampling path and, in turn, a complete gas map with a relatively low number of measurements. Furthermore, it also accounts for wind currents in the environment, which improves the reliability of the final gas map even in the presence of obstacles or when the gas distribution diverges from an ideal gas plume. Finally, we report various simulation experiments to evaluate our proposal against a computer-generated fluid dynamics ground truth, as well as physical experiments in a wind tunnel.

## 1. Introduction

Monitoring and enhancing indoor environmental standards, such as air quality, thermal comfort, or acoustics, are of great importance for improving human health, comfort, and productivity [1]. Due to the complexity of indoor structures typical of human-centered environments, airborne pollutants are not distributed homogeneously, resulting in significant spatio-temporal variations. In this work, we focus on mobile sensing approaches, an interesting and promising alternative to the traditional use of a static network of sensors [2,3] because of their versatility and coverage capabilities.

Mobile robotic olfaction, the discipline that combines artificial olfaction with mobile robotics, often involves applications where a robot has to detect the contour of a gas distribution and measure its intensity [4]. In these situations, the robot’s role is to explore an environment to obtain a map of the gas distribution. These maps could then be an asset, for example, to tailor an automated fire evacuation response depending on how smoke is spreading through a building [5], to determine the origin of a volatile substance during robotic Gas Source Localization (GSL) [6,7], or as an intermediate tool to investigate urban air pollution [8].

Acquiring the necessary data to generate such gas distribution maps is not a trivial task. The phenomena of diffusion and advection often lead gases to spread in a highly stochastic fashion [9], making it very hard or even impossible to accurately predict how gas particles move with dispersion models alone. Moreover, limitations in current gas sensing technologies have to be taken into account. To date, no gas sensor allows capturing a complete gas distribution with a single *snapshot* [10], and visiting every possible location to take a gas sample with an electronic nose (e-nose), for instance, is either very time-consuming or, quite often, intractable if there are places that the robot cannot physically reach. Hence, the prevailing approach for olfaction-enabled mobile robots is to perform a partial exploration of the environment and then extrapolate the gas concentration to non-visited locations. This is accomplished with Gas Distribution Modeling (GDM) [11,12,13], the process that estimates the shape of a gas distribution from a set of sparse measurements that are composed of gas observations (e.g., from the robot’s e-nose [14,15]) and, in some cases, wind data from an anemometer [16,17]. For a detailed review of gas sensing technologies and e-noses commonly used in mobile robotics, please refer to [18,19].

Multiple GDM approaches have been proposed for mobile robotics. Some techniques extrapolate the robot measurements to nearby locations with *Gaussian* kernels [20], others rely on Source Term Estimation (STE) [21] to predict the parameters that govern the shape and origin of a gas plume [22], and others constrain the gas dispersion to the presence of obstacles [23]. Regardless of the mathematical formulation, all GDM techniques for mobile robotics aim to fulfil one requirement: their computation must happen in real time, or as close as possible to it, to provide the robot with a continuously updated gas map that accounts for the latest gas measurements. This is paramount for applications such as industrial safety [24], where fast decision making is necessary and where more accurate Computational Fluid Dynamics (CFD) simulations would be too slow to perform.

One such real-time GDM algorithm is Gas-Wind Gaussian Markov Random Field (GW-GMRF) distribution modeling [25]. This algorithm treats the environment as a lattice and models the interaction between adjacent cells as a probability distribution to estimate the most likely gas concentration at remote locations (see Section 3 for more details). The main advantage of GW-GMRF over similar techniques is that it also accounts for the obstacles in the environment and for the local wind measurements captured by the robot to reduce the total number of samples it has to gather. Moreover, because GW-GMRF combines all gas and wind samples under a probabilistic approach, it can assign an uncertainty level to each location of the output map. This provides valuable feedback to determine how trustworthy the estimates are, which, in turn, allows GW-GMRF to deal with challenging situations, such as physically separated areas (e.g., walls), eddies near obstacles, or environments that are only partially explored, without impairing its overall reliability.

Similar to most GDM techniques, GW-GMRF has one important limitation: it is an open-loop method that provides no feedback on where the robot should explore next; it only takes a set of input samples and computes the gas distribution map that explains them the best. However, the limited time and power available to explore the environment with a robot imply that it is critical to plan sampling locations that yield the most informative sensor readings. The main difficulty herein lies in the lack of a priori knowledge about how the gas is distributed, which prevents the robot from computing the optimal exploration path in advance. A conventional sensing strategy that samples along a predefined path does not take into account the distribution and valuable resources might be wasted at locations of little interest [26]. Certainly, a better strategy would be one where the robot can dynamically adapt to the latest gas distribution estimates and plan the remainder of its exploration accordingly. In the case of GW-GMRF, this can be achieved by resorting to an uncertainty map, whose function is precisely to highlight where the estimate is deficient and more data are needed. However, just visiting the locations with the highest uncertainty in sequence might still lead to erratic and sub-optimal behavior. For example, the robot could end up in an unintended loop where it repeatedly transverses the same areas to reach locations with high uncertainty at opposite ends of the environment.

For situations where a mobile robot needs to acquire information about the environment yet is unable to plan an optimal strategy without this very information [27], a Partially Observable Markov Decision Process (POMDP) can be a possible solution. The POMDP framework was originally conceived for robotic control, dealing with the uncertainty in the robot measurements and the outcome of its actions [28]. In particular, it is useful for non-deterministic scenarios where a robot has to maximize a goal (e.g., get close to a target or collect objects) whilst saving on resources (e.g., energy and execution time). In these cases, the robot is provided with a complete representation of the environment, which is subsequently leveraged to predict the likely outcomes of an action. After implementing some slight modifications, the POMDP framework can also be applied for information gathering tasks [22,29]. For example, when it is applied to GDM, the robot might know the shape of the environment, but not how the gas is distributed within it.

**Figure 1:**
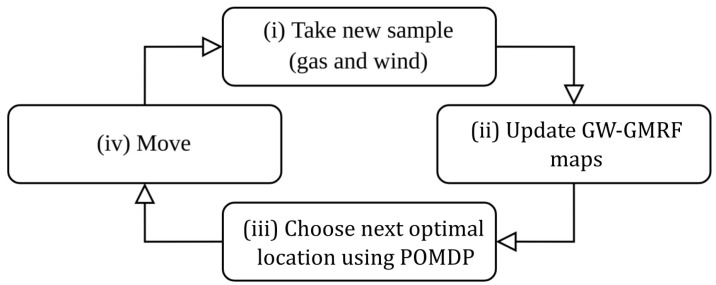
Our proposed control loop.

This paper combines GW-GMRF with a POMDP method to create a closed control loop for autonomous GDM. Although GW-GMRF is compatible with multi-agent systems and sensor networks alike, we will focus on the case of a single mobile robot equipped with an e-nose and a 2D anemometer. Our system’s control loop, as shown in Figure 1, comprises four steps: the robot (i) takes a new set of gas and wind measurements, (ii) updates the corresponding GW-GMRF maps, (iii) exploits the estimated current map to predict the most likely outcome of its future movement options, and (iv) executes the one that yields, a priori, the highest information gain. The role of the POMDP in this loop is to ensure that the robot movements attain the greatest reduction in the uncertainty of GW-GMRFs estimates while moving along the shortest possible path, or, equivalently, to maximize the information added to the gas distribution map after each step. Therefore, we refer to this method as Information-driven Gas Distribution Mapping (IGDM).

Our core contributions are thus as follows:
  - The integration and customization of the POMDP for an efficient exploration of the environment, guiding the robot toward informative points for GDM.
  - A GDM formulation that leverages GW-GMRFs main strength: the ability to operate under complex indoor conditions and in the presence of complex wind patterns.
  - A comprehensive evaluation of IGDM via simulations and real experiments in a wind tunnel, along with a detailed discussion of its strengths and weaknesses.
  - The release of an open-source package of IGDM (https://github.com/MAPIRlab/igdm, accessed on 23 April 2023), including a complete implementation of GW-GMRF (https://github.com/MAPIRlab/gdm, accessed on 23 April 2023) to aid in the development of future research by the community.

## 2. Related Work

Autonomous mapping, understood as an Informative Path Planning (IPP) problem, is an active research topic that centers on optimizing a robot’s exploration path to gather information about a physical phenomenon with the objective of reconstructing its spatial distribution. In this section, we review some of the most relevant publications centered on reconstructing how chemical volatiles distribute in the environment, including alternative GDM methods as well as some precursors to IGDM (our method).

Our work draws inspiration from the work on *infotaxis* [30], a strategy that searches for gases by maximizing the expected information gain. *Infotaxis* relies on an underlying gas dispersion model to compute a prior probability distribution over the environment from past gas measurements. For each incremental step of the exploration, this prior distribution enables the robot to determine the travel direction that should, hypothetically, yield the most informative and valuable samples. The behavior and capabilities of *infotaxis* are, consequently, not static, but determined by the employed gas dispersion model and its fitness for the task at hand. The first version of this strategy [30] and its multi-robot variant [31], for instance, were exclusively aimed at GSL, finding the release point of a target gas. These employed a simple yet effective model of how frequently a robot is expected to encounter gas patches given its distance to the source. This model worked well to track sparse gas plumes, but not to map their spatial distribution.

Another infotactic approach, based on STE [22,32], models the gas distribution as a pseudo-Gaussian plume whose origin coincides with the source and whose concentration can be computed for every point in space (also in 3D). However, it aims to track relatively straight gas plumes under stable wind conditions, and thus may fail to provide reliable gas maps in the presence of obstacles. Our work, on the other hand, takes full advantage of GW-GMRFs ability to deal with complex indoor environments, albeit oriented purely towards infotactic GDM rather than GSL.

An important aspect of IPP approaches is how to determine the informative content and overall importance of each potential sampling site. In applications that involve gases [26,33], this is usually accomplished by computing the Kullback–Leibler Divergence (KLD) between the prior distribution and the later one that would be obtained after taking measurements at each candidate goal position. However, just choosing the sampling sites that yield the most information alone would not lead to a sensible exploration of the environment. The robot must also take into account the time and effort it takes to reach them, and if there are multiple sites of interest, in what order they should be visited. One possible tool to determine such a (pseudo) optimal path could be a POMDP formulation (more details in Section 4). A POMDP simulates the outcome of every possible movement and then picks the one with the best score according to a given reward function, so that it may also account for other relevant parameters (travel time, distance, and energy cost) aside from the KLD gain [34,35].

Regarding computational efficiency, POMDPs and similar IPP methods are usually constrained by the fact that the robot may only choose from a finite set of actions, which have to be checked individually. Efficient alternatives have been proposed for Gaussian Markov random field (GMRF)-based IPP [36,37] exploiting their closed-form solution. However, GW-GMRF introduces a dependency between a gas map (a scalar field) and a wind map (a vector field) that must be solved numerically, and avoids such implementations.

## 3. GW-GMRF Gas Distribution Mapper

To keep this manuscript self-contained, we will start by reviewing the main characteristics of the GW-GMRF algorithm [25]. GW-GMRF is a 2D gdm method that combines gas and wind measurements to account for advection, i.e., the effect by which gas molecules are carried in the general direction of the wind [38]. To do so, GW-GMRF leverages the robot’s knowledge about obstacles in the environment (usually its navigation map) to simultaneously model how the gas distribution and wind currents spread out and interact with each other. This allows GW-GMRF to compute far-reaching yet robust estimates for both magnitudes even when the robot has gathered only a few observations or is operating in complex indoor environments.

As its name implies, GW-GMRF is formulated as a Gaussian Markov random field, a grid map whose cells encode the gas and wind values as Gaussian variables. Although modeling the gas concentration with a Gaussian distribution may lead to the consideration of negative concentration values with non-zero probability, a fact that has no physical meaning, it leads nonetheless to an efficient solution that works well in practice, as shown experimentally. To attain an efficient solution, GW-GMRF only models the relationships between neighboring cells [25], computing the grid map that best satisfies all rules at a given time step, interpreted as a least-squares problem [39]. For example, if there are no obstacles between two adjacent cells, they must have similar gas and wind values (i.e., no abrupt changes allowed). Additionally, if wind enters a cell from any side, then it must leave through any of the others (i.e., the total air mass is conserved) and may not flow if there is no exit for it. Moreover, the gas concentration between neighbors may not significantly change in the direction of the wind (i.e., advection causes gas plumes). The robot’s measurements are also taken into account by constraining that the value of any measured cell must be very close to the corresponding sensor reading, but may not be necessarily the same, accounting for sensor noise, contradictory inputs, and the diminishing relevance of old samples (i.e., the measured value of a cell may change when sampled repeatedly).

**Figure 2:**
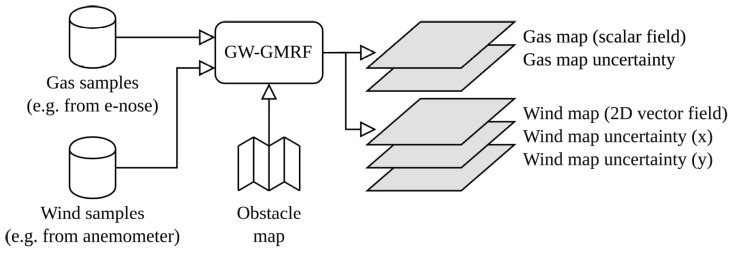
Functional diagram of the GW-GMRF algorithm. It takes as inputs a set of gas and wind samples from known positions, usually from an e-nose and anemometer carried by a robot, as well as a map of the environment that shows the presence of obstacles and possible wind inlets and outlets. The outputs are the estimated gas and wind maps and their associated uncertainties, represented as Gaussian distributions on a grid map.

As shown in Figure 2, the input of GW-GMRF is a set of gas and wind samples gathered at known positions. Since the grid map represents the gas and wind estimations in each cell as Gaussians, the output can be separated into five maps for convenience. These maps represent the estimated mean value and uncertainty (standard deviation) of the gas concentration and the wind components in the X and Y directions. Notice that the mean value of the wind flow is provided as a vector field. These estimates and uncertainties are, however, not computed simultaneously. GW-GMRF first produces the gas and wind estimates, with a computational complexity of $O(3n^{2})$ for a map with *n* cells, and then, separately from each other, the gas uncertainty map ($O(n^{6})$) and the wind uncertainty maps in both the X and Y directions ($O(8n^{6})$).

Another consideration in this regard is that the output of the GW-GMRF algorithm has a fixed, albeit user-configurable, resolution. Usually, smaller cells are desirable to obtain more accurate estimates. However, a trade-off between resolution and computational complexity is necessary, usually taking into account the dimensions of the robotic platform and the environment [23,25].

GW-GMRF has a total of 11 control parameters, including the aforementioned cell size, how fast the robot’s observations age and lose relevance, and the relative weights of all the cell connectivity rules. These parameters can be adjusted depending on the target application and its requirements, for example, to limit the influence of older samples or to fine tune the accuracy of the sensor input. In this work, we chose the parametric values proposed by Gongora et al. [25] for generic indoor scenarios, except for the cell size, the value of which is dynamically altered in Section 5 to attain a more efficient implementation.

## 4. POMDP Formulation

The function of a generic POMDP, composed of {S,A,T,R,Ω,O} and applicable to control problems in general, is to determine a (pseudo) optimal sequential control policy for an agent in a non-deterministic system. It maintains a belief distribution *s* over all possible states *S* that the agent can be in. At every iteration, out of the set of possible actions A:S→S, it chooses the one that maximizes its reward function $R(s,s^{\prime })$. The reward function is a user-defined metric describing how well the agent is fulfilling its goal and how many resources (e.g., energy and time) its action would likely require. Additionally, the POMDP must account for the state transition probability T:S×A→Π(S) of the actions over the probability distribution of possible states Π(S), since arriving at state $s^{\prime }$ that is different from the intended one might offset the reward in favor of other options. Lastly, the set of all possible observations Ω, distributed according to O:S×A→Π(O), where Π(O) denotes the probability distribution, participates after the action has been completed and the POMDP updates its state belief before the next iteration.

A POMDP can be an efficient strategy for situations where the state space *S* and the number of possible observations Ω are both finite and modest in number [29]. However, neither condition applies in our case. Consider, for instance, all the possible gas distributions that this map could eventually represent. The number of variables it will need is enormous, even if the value of each cell is discrete, and so will be the set of all observations the robot could eventually gather, which encompasses all possible combinations of e-nose and anemometer readings for all locations in the environment. However, by limiting its scope, both temporal and spatial, it can be reformulated as an information gathering task [29]; a limited variation of the POMDP that is specifically meant to gather information in unknown environments.

### 4.1. Formulation as an Information Gathering Task

Instead of solving for an analytical solution, information gathering POMDPs rely on *forward simulation* to approximate the outcome of the actions on a case-by-case basis. In this way, the POMDP no longer has to consider all possible scenarios, but can only focus on those that are actually relevant at any given time. In our case, the accuracy of the GW-GMRF gas map improves depending on where the robot takes the next measurements by sequentially choosing the option with the highest projected reward given its latest belief about the gas distribution.

The drawback of this approach, however, is that the robot may no longer move in just any direction. Since it has to simulate the outcome of all available movements for every new state belief, after updating its GW-GMRF map with the previous measurement, the problem now becomes NP complete [40]. Usually, this can be alleviated through Monte Carlo-based approximations [22,41]. However, due to the already elevated computational cost of GW-GMRF, a reasonable alternative is to limit the robot’s movements to a few discrete options that are computationally affordable to check.

The POMDP for IGDM is formulated with only three elements: its belief distribution, the set of actions the robot may take, and the reward function that governs the exploration. Once all three are defined, we can solve our problem through forward simulation, as explained in Section 4.2.

#### 4.1.1. IGDMs State Space Belief, *s*

We make use of GW-GMRFs latest estimated gas map as the belief distribution *s* over the environment. The actual gas distribution cannot be directly observed; thus, this represents the IGDM algorithm’s best estimate of the ground truth and therefore serves to predict the values the robot would measure when evaluating its movement options for the next step.

#### 4.1.2. IGDMs Movement Actions, *A*

Instead of considering the full range of motion, IGDM limits the robot’s movements to the underlying GW-GMRF grid map one axis at a time. Accounting for positive and negative movements with a fixed distance $d_{A}$, this results in a total of |A|=4 possible movements (N, W, S, and E). This allows IGDM to fully explore the environment and, as long as $d_{A}$ is selected in proportion to the environment geometry, to circumvent all obstacles. It must be noted that $d_{A}$ is the maximum step distance. If IGDM detects that a step would lead to a collision on the robot’s obstacle map, it might shorten the distance accordingly.

#### 4.1.3. IGDMs Reward Function, *R*

The reward function determines how IGDM chooses the next movement by assigning a numeric *profit score* to each possible goal position. Ideally, it should favor movements that lead to sample locations with useful information, whilst providing little incentive for those that were recently visited. Since GW-GMRFs gas uncertainty map can only improve with new samples, a suitable reward function would be one that assigns better scores for greater reductions in uncertainty.

We measure this change as the KLD between GW-GMRFs current gas estimate and the one it should produce after acquiring new data. Naturally, there is no way to anticipate the exact gas and wind values that will be observed, but, for relatively short steps, these can be predicted from GW-GMRF maps. It must be noted that GW-GMRF maps are intrinsically multivariate Gaussian distributions and therefore handle the uncertainty on the estimated gas concentration or wind vector, respectively (see [25] for more details). Once the new estimate is then forward simulated, we can compare its gas map to the current one with the following reward function:

(1) $$R_{KLD}\left(a|s\right)=\mathrm{KLD}\left(N\left(m_{g_{s}},\Sigma_{g_{s}}\right),\;N\left(m_{g_{a}},\Sigma_{g_{a}}\right)\right)$$

which measures the information gain between the current gas estimate $g_{s}$ computed for the real samples and the expected estimate $g_{a}$ after movement *a* predicted during forward simulation, both expressed as multivariate *Gaussian* distributions, since they stem from GW-GMRF estimates.

To capitalize on the physical correlation between wind flow and gas dispersion (e.g., if a cell contains gas, we can extrapolate gas presence in the downwind direction), the reward function *R* should prioritize actions that drive the robot to measure new areas with expected gas presence. Thus, to balance the robot’s tendency to map the gas it has already detected against its other tendency to explore new areas, we scale the reward function by the following component:

(2) $$R_{g}\left(a|s\right)=\tanh\left(k_{g}\int_{p_{a}}g_{s}\;d_{p_{a}}\right)$$

where the integral denotes the accumulated gas that is expected (according to the latest state belief) along the segment of path $p_{a}$ that the robot would explore if it were to execute action *a*, treated as the input of a *sigmoid function* (in our implementation a hyperbolic tangent tanh) that is 0 if no gas is measured and saturates at a value of 1, meant to prevent locations with extreme gas concentrations from eclipsing those with more moderate values. The parameter $k_{g}$ controls the saturation rate and can be adjusted depending on the importance of the gas concentration; small values lead to little saturation whereas higher ones cause $R_{g}\left(a\right)$ to behave in a binary fashion, where the reward will be 0 if there is no gas and 1 if the presence of gas is detected.

Lastly, the reward function should also minimize the amount of energy and time spent during exploration, which, depending on the particular configuration of the robot, usually means minimizing the length of the path. For a generic robot, the simplest option is to penalize each action according to:

(3) $$E_{m}\left(a\right)=k_{d}d\left(a\right)+k_{\theta }\theta \left(a\right)$$

where d(a) and θ(a) denote, respectively, how much distance the robot would advance and rotate for any given action and $k_{d}$ and $k_{\theta }$ control their respective unit costs.

Combining all terms together in an equivalent way, similar to [42], we obtain the following reward function, where the information gain $R_{i}\left(a\right)$ is scaled up depending on the detected gas component $R_{g}\left(a\right)$ and its relative weight $\epsilon_{g}$, and scaled down by the movement energy $E_{m}\left(a\right)$:

(4) $$R\left(a|s\right)=\frac{R_{KLD}\left(a|s\right)\left(1+\epsilon_{g}R_{g}\left(a|s\right)\right)}{E_{m}\left(a\right)}$$

Please note that the final reward function does not demand computing the wind uncertainty at any stage. Our goal only entails minimizing GW-GMRFs gas uncertainty when mapping out the environment. This does not, however, prevent the robot from computing said uncertainty if it were to need it for other purposes, but it allows us to skip GW-GMRFs computationally most expensive part when computing the reward for all action sequences in this specific implementation.

### 4.2. Finite Horizon Forward Simulation

In forward simulation, the robot has to evaluate the reward function for all possible actions before it can pick the best one. This involves predicting the observations each future action will presumably yield, followed by simulating how they would impact the current belief state and finally choosing the most promising one. When forward simulation is applied for information gathering tasks where the robot has no prior knowledge about the environment, the real outcome of any given action can differ from its expectation. It makes therefore little sense to plan multiple actions ahead of time while their true effects cannot be predicted, and as a result, action planning in these cases is usually restricted to a *greedy exploration* that only considers the immediate best action [29].

In the case of this work, however, since GW-GMRF is assumed to have a relatively complete map of the environment (usually the robot’s navigation map), some prior knowledge about the environment exists. This means that, even though we do not know how the gas is distributed, we can still identify movements that lead to areas with high uncertainty or where advection should lead to strong extrapolation. After all, the disposition of walls and obstacles constrains how the gas and air currents distribute, which, in turn, limits the cumulative error between sequential predictions. Therefore, by constraining GW-GMRFs estimate as well as the available actions to the obstacle map, we may leverage a multi-step action plan during forward simulation.

**Figure 3:**
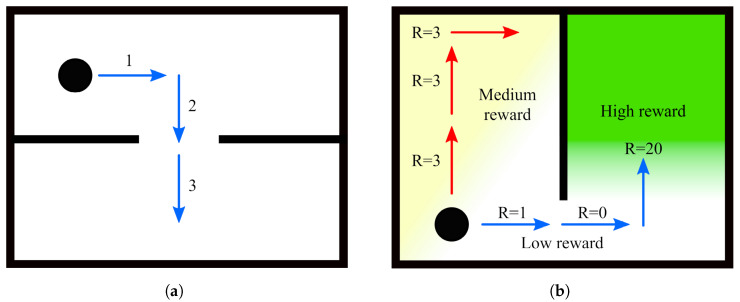
Illustration of multi-step path planning. Although each movement step is in a straight line, they can be combined in sequence to assess the reward for a path that (**a**) bends around corners and obstacles in complex environments or (**b**) can reach high reward areas that would otherwise be blocked off by a low reward area. In this latter example, if the robot were to plan for one step only, it would stay in the medium reward area (a reward of 3 instead of 1), but by planning for several steps, it realizes that the blue path has a better total reward than the red one and should thus be preferred (a total reward of 21 instead of 9).

The advantage of planning several steps into the future is that it allows the robot to cope with more challenging situations, such as planning a path around corners (Figure 3a) or reaching locations that are separated by already explored areas (Figure 3b). Note that although several actions are planned, only the first in the sequence is executed, after which the robot reevaluates its state belief and updates the action plan accordingly. Thus, under this *finite horizon* action planning, the goal of IGDM becomes to determine the sequence of movements *M* composed of a combination of actions from *A* that maximizes the total expected reward for *H* consecutive steps:

(5) $$M=\underset{(a;a_{1},\ldots ,a_{H})\in A}{\mathrm{argmax}}\;\sum_{i=1}^{H}\gamma^{i-1}\;R\left(a_{i}|s_{i}\right)$$

where $s_{i}$ is the state belief after the previous action ($s_{i=1}$ is the current GW-GMRF estimate) and $\gamma^{i-1}$ is a discount function that diminishes the reward of later movements to account for their less certain outcome, which in our case is an exponential with constant base γ≤1 so that it evaluates to 1 for i=1.

One important remark about the search of all possible sequences for *M* is that all combinations must be evaluated independently and do not benefit from techniques such as Monte Carlo Tree Search (MCTS) [43] or variants of the Rapidly Exploring Random Tree (RRT) [44]. The reason for this limitation lies in the fact that the reward of a given action $a_{i}$ is not correlated with the reward of its preceding action $a_{i-1}$, yet it depends on the state $s_{i}$ said former action would have led to. This means that sequences that start with an action that yields a low initial reward, and would thus be discarded by the aforementioned techniques, may actually branch out to the path that has the highest final reward, such as in the example of Figure 3b. Nonetheless, there are still some performance gains that can be easily obtained to accelerate the search and which we discuss in the next section, as well as other heuristics that could be exploited in future works.

## 5. Implementation and Parameter Selection

In this section, we go through different implementation aspects that have been considered along the different steps that compose the proposed IGDM method (see Figure 1) to produce a reliable and efficient code.

**Figure 4:**
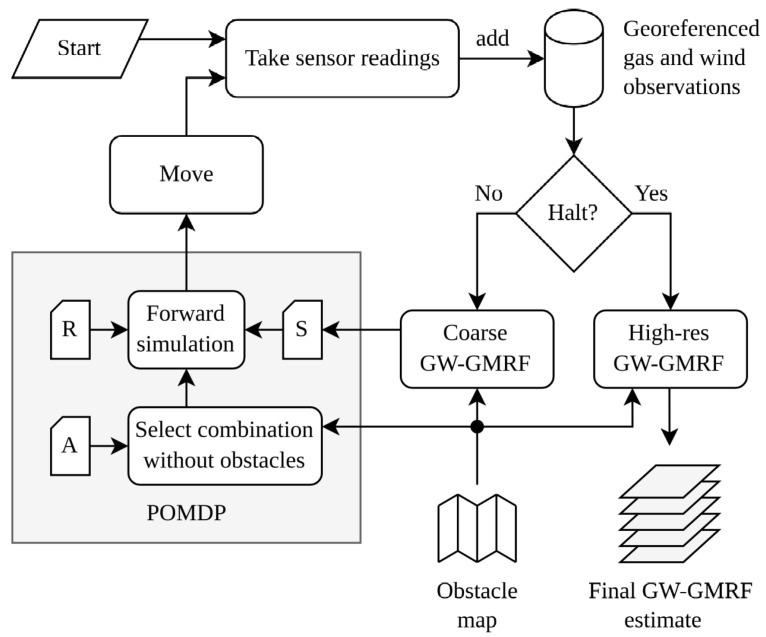
Control loop for IGDM. After acquiring the first gas and wind samples and as long as the halting criterion is not met, IGDM estimates the gas map with a coarse (low-resolution) GW-GMRF estimator. This map is then fed into the POMDP to simulate the outcome of the reward function *R* for the possible movement combinations in *A* (accounting for obstacles using the robot’s obstacle map) and choose the optimal movement path. After, or while, executing the first action in said path, the robot takes new sensor readings and repeats the process. All gas and wind observations are stored along with information of when and where they were collected, so that IGDM may produce a high-resolution GW-GMRF estimate at the end of the exploration or (although omitted in the illustration) at any other moment the robot might need the latest maps.

As shown in Figure 4, the implementation steps comprise a coarse fine GW-GMRF estimator, the limitation of the robot’s possible motion actions, the definition of the halting criteria, and the review of the main parameters.

### 5.1. Coarse and Fine GW-GMRF

The main drawback of the forward simulation mode is its computational cost during run time. Every time the robot wants to choose a movement, it first has to compute separate GW-GMRF estimates to compare the outcome of all available options. If we consider that GW-GMRF is already a relatively slow gdm method, then running it tens or potentially hundreds of times for every iteration might prevent IGDM from working in real time, especially on less resourceful robots.

The simplest solution to this problem is to reduce the computation time of GW-GMRF. As we saw in Section 3, this can be easily achieved by lowering the resolution of the estimated maps because the computational complexity is, for an environment of a given size, proportional to the total number of cells *n* that must be computed, i.e., O(n). For instance, a cell size of around 1 m works for medium-sized settings remarkably well in practice and offers an acceleration factor over a cell size of 0.1 m of around $\frac{n_{\mathrm{coarse}}}{n_{\mathrm{fine}}}=\frac{1^{2}}{0.1^{2}}=100$. However, increasing the cell size leads to much coarser estimates that might no longer satisfy the requirements of the application needing the gas map. Thus, we suggest adding a second GW-GMRF estimator in parallel that offers the possibility to produce gas maps with a finer resolution that are only computed on demand (e.g., the robot might periodically compute it in parallel to the main loop) or once the IGDM process is finished (more on this in Section 5.3), as proposed in the previous literature [45].

### 5.2. POMDP and Forward Simulation

The POMDP decision block in Figure 4 is implemented as described in Section 4. For each iteration of the control loop, it treats the coarse GW-GMRF gas estimate as the current state belief *s* to predict the outcome of the various movement combinations from *A* over a finite horizon of *H* steps. It then evaluates every option according to the reward function *R* and, finally, instructs the robot to execute the movement in the sequence that is projected to achieve the highest reward.

**Figure 5:**
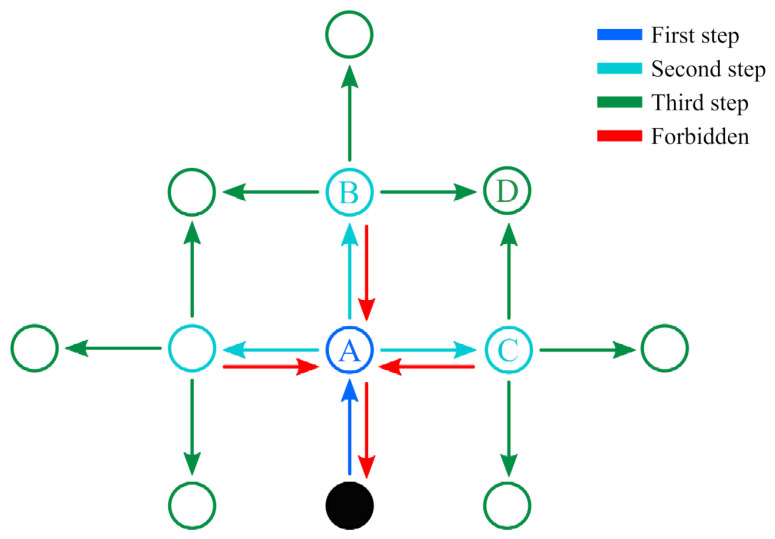
Example of the possible movement options for the robot. The current position is the filled circle and the first movement is assumed to be North (A). After reaching position A, the robot may move in any direction except South to avoid backtracking (red arrows) and revisiting the same location twice. The result of limiting the movement options is that the number of possible combinations to be checked is reduced. Note that some positions are reachable over different paths (e.g., D can be reached either over B or C), that might have a different POMDP reward and/or avoid obstacles.

To further alleviate the computational requirements of IGDM, only part of the possible motion sequences are considered. Instead of evaluating absolutely all |A| combinations of the available actions, which would consequently require ${\left|A\right|}^{H}$ GW-GMRF estimates for *H* consecutive steps, we only account for those sequences where the robot does not backtrack. In other words, we only evaluate the options where the robot does not undo any of its movements, as shown in Figure 5. While planning the trajectory, moving immediately to a position that has just been sampled provides less information than any of the alternatives because the forward simulation will use the same concentration map both times. Moreover, we can disregard trajectories that loop back to repeated positions, as they also provide less information than those that stretch over a wider area. As a result, IGDM only has to consider $\left|A\right|{\left(|A|-1\right)}^{H-1}$, which for |A|=4 movement options and a horizon of 3 steps, for instance, is a reduction from 64 to 36 combinations that have to be evaluated.

We must remark that the robot’s obstacle map is always taken into account. If any action would lead the robot into an obstacle, then the step size is shortened as needed or, when too short, completely disregarded to further save computation. This also ensures that the energy component of the reward function is properly accounted for.

### 5.3. Halting Criteria

IGDM would, under the formulation we have presented so far, continue to explore the environment indefinitely. This might be desirable for scenarios where the gas distribution is prone to continuous changes and must be resampled periodically. However, if the application requires the robot to eventually finish, then a halting criterion must be defined. This could entail, for example, stopping after the global uncertainty falls below a threshold or after the robot has spent a fixed amount of time exploring. Another option can be to add a wait action for the robot to consider, so that it can periodically rest (or dock at a charging station) while it waits for the uncertainty of the map to slowly rise when GW-GMRF disregards old samples. As illustrated in Figure 4, our implementation uses a simple and fixed limit that is based on the total length the robot has traveled. Once such a limit has been reached, the robot stops and computes the final maps. The exact criterion and implementation of the halt condition can be changed for other applications of IGDM.

### 5.4. IGDM Parameters

The behavior of the IGDM algorithm is influenced by the parameters of its different parts, namely POMDP and GW-GMRF. Since the choice of the parameter values depends on the target application [16,23], we selected the parameters of GW-GMRF according to those suggested by the authors for generic indoor scenarios in [25] and then adjusted the ones of our POMDP with a balanced [46] stochastic gradient descent for the same indoor conditions. With respect to the latter, we wanted a combination of the scaling weights in IGDMs reward function (γ, $\epsilon_{g}$, $k_{g}$, $k_{d}$, and $k_{\theta }$) that led to the greatest reduction in the root mean squared error (RMSE) between its estimated gas map and the ground truth of simulated training environments for a given maximum length of the exploration path. We ran several hundred simulations with different initial conditions and indoor environments to locate a combination in the parameter space that would perform reliably for all test conditions. The values we have obtained in this way seem to work well in practice, as demonstrated in Section 6, but they are not guaranteed to be optimal given how they have been obtained. Developing an efficient method to set IGDM parameters for a given application scenario falls out of the scope of this work.

Our final selection of the parameters is shown in Table 1. According to our experimental results presented in the following section, these parameters lead to reasonable exploration paths under very diverse conditions. Nonetheless, we must stress that they are very application dependent and should be chosen carefully when implementing the IGDM algorithm.

## 6. Experiments and Discussion

This section describes the experiments carried out using a wheeled mobile robot running the IGDM algorithm. We first present a set of simulations where the ground truth is available and the algorithm’s performance can be measured precisely. Then, we report on physical experiments carried out in a wind tunnel, where we focused on the exploration’s consistency for a realistic gas plume as well as a gas distribution distorted by obstacles. Lastly, we discuss the performance of the algorithm to provide a better insight into its capabilities and justify some of the implementation decisions from the previous section. Please note that, unless otherwise specified, all parameters were set as described in the previous section.

### 6.1. Simulation Experiments

The simulation experiments are intended to validate the IGDM algorithm under controlled and repeatable conditions. Next, we specify the setup and tools employed for the generation of these experiments.

**Figure 6:**
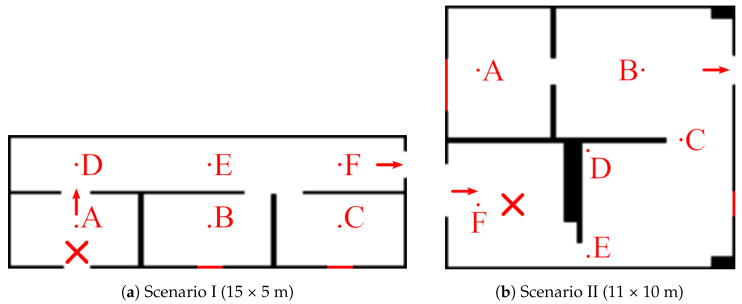
Floor plans of the simulated scenarios. Red crosses depict the locations of the gas source (release point), and labels A–F mark the starting points for the robot. Only one inlet /outlet in each scenario is left open, while the others are marked in red.

**Figure 7:**
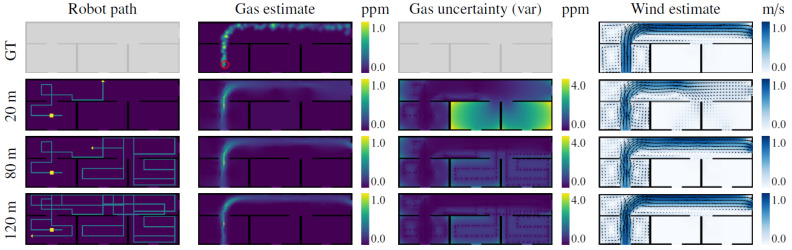
IGDM exploration of scenario I showing the ground truth (top row) and the estimate after 20, 80, and 120 m of exploration. On the robot path map, the starting position is denoted by a yellow square and its positions in the gas and wind maps are estimated by an arrow. The gas source is shown as a red circle in the ground truth.

**Figure 8:**
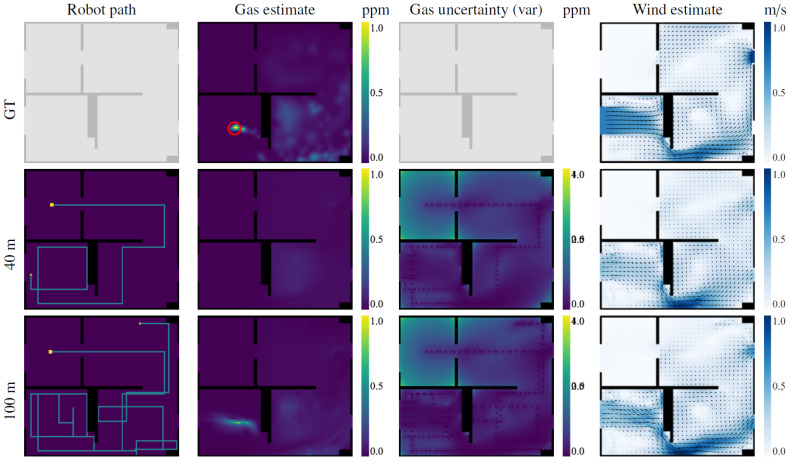
IGDM exploration of scenario II showing the ground truth (top row) and the estimate after 40 and 100 m of exploration. On the robot path map, the starting position is denoted by a yellow square, and its positions in the gas and wind maps are estimated by an arrow. The gas source is shown as a red circle in the ground truth.

**Figure 9:**
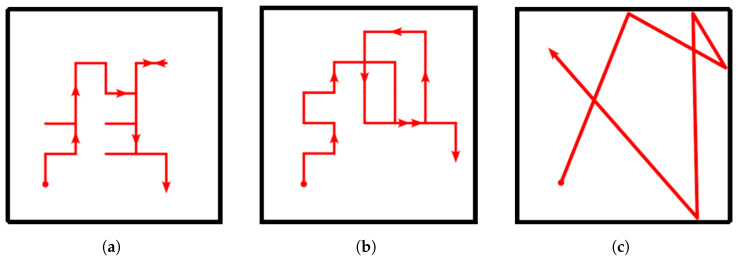
Example path for the random exploration strategies. (**a**) Brownian strategy that selects uniformly between movements in the N, W, S, or E direction with the same step size as IGDM. (**b**) Similar to (**a**) but undoing the last movement is forbidden. (**c**) Advance until collision strategy, turning a random angle when an obstacle is reached.

- **Scenarios:** We considered two test scenarios, shown in Figure 6, that were selected to represent situations a robot might encounter in a real indoor environment. On the one hand, Scenario I depicts a long corridor with adjacent rooms where we simulated a 2D quasi-ideal gas plume (i.e., continuous and with straight sections). On the other hand, Scenario II presents a more challenging scenario depicting an office-like environment, simulated in 3D, that leads to a very diffuse gas distribution with many discontinuities. A snapshot of the simulated plumes and both gas concentration and wind vector maps can be seen in Figure 7 (top) and Figure 8 (top), respectively.
- **Wind Flow Simulation**: The wind flow in each scenario was simulated with OpenFOAM [47], a realistic CFD toolbox. The wind conditions were determined by closing all inlets and outlets except two: one that acts as a wind inlet at 1 m/s (typical of indoor scenarios [48]) and another that serves as an isobaric outlet. It is important to note that during the mapping process, the robot knows the location of the inlets/outlets in the environment, but does not know if they are open or closed, which needs to be learned from the measurements during execution.
- **Gas Dispersion Simulation:** For the gas dispersal, we relied on the open-access gas dispersion simulator GADEN [49]. The gas distribution was simulated for a single, fixed gas source, consisting of an ethanol gas release at an approximate rate of 100 ppm/sec. All simulations were conducted in continuous time, which means both wind and gas fields contain dynamic eddies and gas patches, respectively. We allowed a 30 second time window before starting experiments to ensure that the transient response had settled, and guarantee impartial conditions with respect to the robot’s starting position. The gas concentration of both scenarios was also normalized for convenience and easier comparison between their distributions.
- **Simulated Robot and Sensors:** To attain similar conditions to the real experimental setting presented in Section 6.2, we simulated a differential drive wheeled robot, equipped with a chemical sensor and a 2D anemometer, sampling at 2 Hz. The robot was programmed to move at 0.5 m/s with a turn rate of $\frac{\pi }{4}$ rad/s.
- **Performance Comparison:** Since the performance of GW-GMRF has been validated separately in a previous work [25], we will focus on analyzing how well the path chosen by POMDP fares when compared against different randomized navigation techniques. These are a Brownian exploration where the robot chooses between the same four movements as IGDM (N, W, S, E), a Brownian exploration without back tracking (i.e., movements that lead to the immediate last position are not considered), and a strategy where the robot advances until it collides with an obstacle and then turns randomly in a new direction. An illustration of these strategies is shown in Figure 9. For the sake of fairness, all strategies will be evaluated with identical conditions to ensure comparable results between our method, IGDM, and the estimates that GW-GMRF would yield when sampling along either of the random paths.
- **Repetitions:** To account for the effect of randomness and to obtain a more meaningful representation of the possible outcomes, we ran all experiments 12 times for each starting robot position (A to F), leading to 72 repetitions for each scenario.

#### 6.1.1. Scenario I: Results

Figure 7 shows multiple snapshots of the gas concentration and wind vector estimates after different exploration lengths for one run of the simulated experiment. It can be seen how during the first 20 m of exploration, the robot moves mostly perpendicular to the wind and wanders out of the room. This provides enough information to estimate the general shape of the gas plume, but it is still not enough to determine whether there is more than one exit for the wind, and whether there is gas in the other rooms as well. The robot then inspects each room until the uncertainties therein drop to the point where the IGDM reward function favors re-inspection of areas expected to contain gas. At this point, after 80 m of exploration, the environment is mostly explored and the gas and wind estimates are very close to the ground truth. If the robot is allowed to continue its exploration, it will also visit some of the locations that were only extrapolated and not sampled, including the center of the main corridor and the lower part of the first room, as shown for the 120 m path.

**Figure 10:**
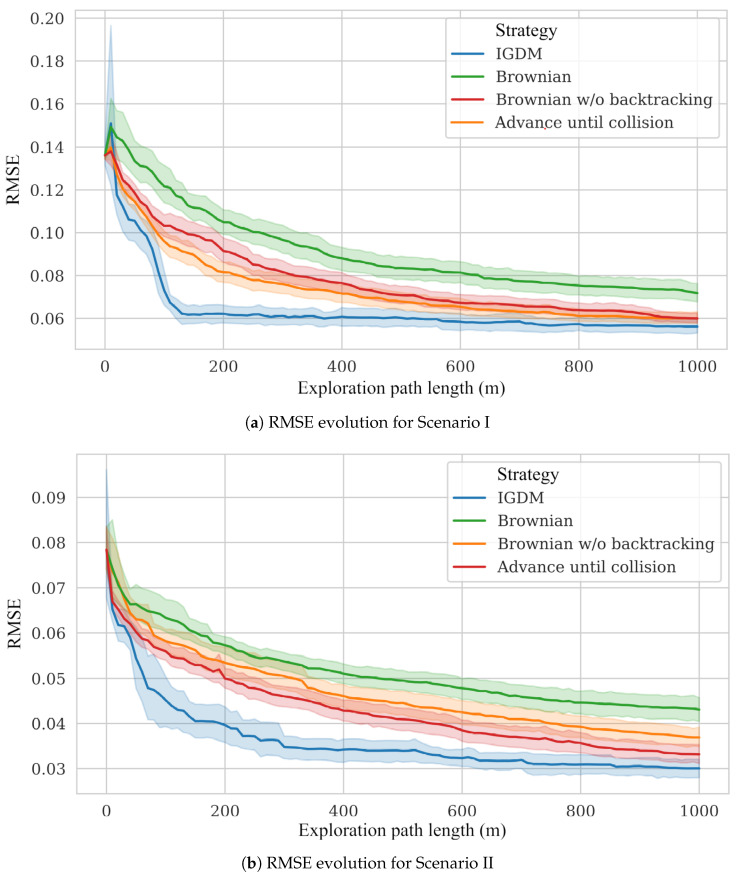
Comparison of the RMSE between the estimated gas map and the simulation ground truth with respect to the length of the robot’s exploration path. Each line represents the average RMSE obtained for the six starting positions (A–F) in each scenario, and the colored area around them represents one standard deviation.

For comparison with the other movement strategies, we computed the RMSE between the estimated gas maps and the ground truth. As depicted in Figure 10a, IGDM outperforms all of the randomized navigation strategies, as it prioritizes the areas that provide the highest reduction in uncertainty. The difference is so pronounced that, after about 150 m of exploration, IGDM achieves the same RMSE that other methods achieve after over 600 m.

#### 6.1.2. Scenario II: Results

The robot’s behavior in scenario II, depicted in Figure 8, is very similar to the one in Scenario I despite the differences in the gas distribution. At the beginning of the exploration, while the robot senses no gas, it tries to minimize the overall uncertainty of the map with the shortest possible path. This behavior is best appreciated during the first 40 m, where the robot travels between rooms in a very straight fashion. However, once the robot detects gas in the lower-left room, its path starts to spiral around the gas source. This simulation was conducted in 3D; thus, most of the gas lies either above or below the plane at which the robot is sampling and consequently is harder to detect. Eventually, after crossing the room several times, the robot detects a gas patch and updates its estimation, and after 100 m of exploration, it will also have mapped the distribution in the lower-right room.

When compared to the other navigation strategies, IGDM shows again a faster reduction in the RMSE (as shown in Figure 10b). In this scenario, given the intermittency of the gas plume, the decay in the RMSE is not as steep as in scenario I.

### 6.2. Physical Experiments

Physical experiments were conducted in a wind tunnel with a volume of $18\times 4\times 1.9\;m^{3}$, generating a constant wind speed of 1 m/s. The wind tunnel was constructed so that the airflow is first conveyed through a flow straightener consisting of tubes of 10 cm diameter and assembled into a honeycomb structure. This leads to a Reynolds number that lies between 4000 and 6000 for the considered wind speed at 15 °C. Afterwards, the wind flow is further laminated through a very fine-structured filter, becoming quasi-laminar right before it enters the main chamber where the experiments were carried out. Here, we placed an electric pump that vaporized ethanol in the air and created a gas plume in the downwind direction. Ethanol was employed for these experiments because it is safe to handle without special equipment, the quantities dispersed are not toxic for humans, and it can be easily removed between experiments by venting the wind tunnel for a few minutes. Moreover, ethanol is heavier than air and stays close to the floor, which makes it easier for wheeled ground robots to detect it.

**Figure 11:**
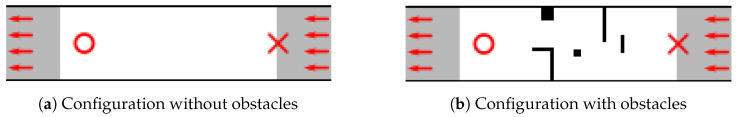
Maps of the wind tunnel where the physical experiments were conducted (**a**) without and (**b**) with obstacles. The length of the tunnel was 18 m, but the experimental area (depicted in white) where the robot can move was limited to 12 m to allow for safety and service areas on each side (highlighted in gray). The wind speed was 1 m/s from right to left as indicated by the arrows, the gas source was located at the border of the experimental area at the position marked with a cross, and the robot started close to the entrance of the tunnel, marked with a circle.

**Figure 12:**
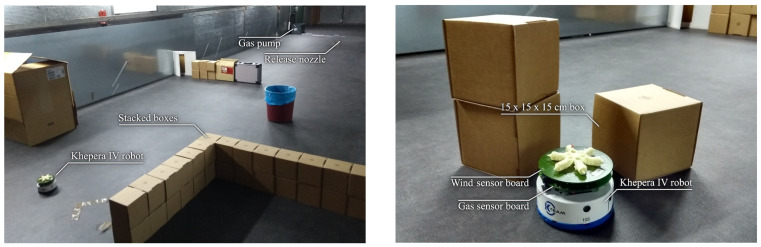
Photos of the experimental setup in the wind tunnel. (**left**) The obstacle course was built with stacked cardboard boxes to block the gas from the emission point (the obstacles in front of it were moved aside for the picture). (**right**) Picture of the Khepera IV robot equipped with a gas and wind sensor board, next to some obstacles for a size comparison.

We repeated the physical experiments under two different conditions to allow for a qualitative comparison with the simulation results described above. The first test was conducted in the 10 m long central section of the tunnel (see Figure 11a) so that the resulting ethanol distribution would approximately be a straight and continuous plume. Then, for the second test, we added an obstacle course that would disrupt the plume and represent a generic indoor environment (see Figure 11b). Although the stacked boxes we used were only 30 cm high, as depicted in Figure 12, we did not measure significant concentrations of ethanol above them with our testing equipment. Running the experiment in a wind tunnel compensated for the lack of an explicit ground truth such as the one we had during simulation. We were able to control, to some extent, the gas distribution based on the disposition of obstacles as well as reproduce almost identical test conditions since the wind speed and gas release point were always the same. We always placed the gas source outside the robot’s test area (see Figure 11) to prevent the robot from getting tangled up in the outlet pipe of the pump.

As shown in Figure 12, the robot used for the experiments was the Khepera IV, a small, 14 cm wide differential drive robot equipped with a MiCS-5521 CO/VOC gas sensor by SGX Sensortech as well as a custom-made wind sensor board [50]. Robot localization was achieved using overhead cameras placed on the ceiling of the wind tunnel, as well as the SwisTrack 4.2 software [51] (https://github.com/d28b/swistrack, accessed on 23 April 2023) running on a separate desktop computer to detect the active markers on the robot. Since the robot’s resources were limited for the purpose of this algorithm, all gas and wind measurements were also relayed to the same desktop computer. After receiving the sensor data, the algorithm first processes the gas sensor readings to mitigate the slow recovery of the employed gas sensor [52] when the robot moves between areas of different concentration, then updates the GW-GMRF estimate and finally computes the next movement command to be sent to the robot. This process is repeated every time the robot complements its movement and sends its new sensor readings.

**Figure 13:**
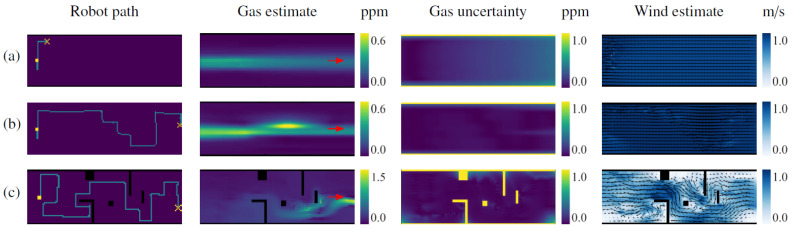
Results of the physical experiments in (**a**) the empty wind tunnel after 5 m and (**b**) after 25 m of exploration, and (**c**) the wind tunnel with obstacles. The robot’s starting position is highlighted as a yellow square, and its position when the estimates were computed is highlighted with a cross. The red arrows on the estimated gas maps indicate the side of the wind tunnel (outside the experiment area) where the gas source was placed.

Although there was no ground truth for the gas and wind maps in the physical experiments, the IGDM algorithm’s behavior appeared appropriate for the selected test conditions. In fact, in the test without obstacles, the robot started by moving perpendicular to the wind, as this provides the highest amount of information. In doing so, the robot was able to estimate the general shape of the gas plume and the complete wind map after only 5 m of exploration, as shown in Figure 13a. The remainder of the path (Figure 13b) consists of upwind crossing from one side of the room to the other. We observed a similar behavior when obstacles were placed in the wind tunnel, as shown in Figure 13c. However, this time, the robot performed a more thorough exploration, focusing more on areas that were shielded from the wind (e.g., the inverted “L” shape), since those areas are harder to extrapolate without wind information. After 40 m, the robot passed in front of the gas source and the estimated gas map was completed.

The results obtained in the real experiments highlight that the behavior of the robot is consistent to the one observed in simulations. Therefore, we can conclude that the IGDM algorithm successfully achieves its purpose in real-world scenarios as well.

## 7. Conclusions and Outlook

In this paper, we presented IGDM, an information-driven algorithm to map complex gas distributions with an autonomous mobile robot. IGDM relies on GW-GMRF to estimate a gas map of the robot’s surroundings from a sparse set of gas and wind measurements, and on an information-gathering POMDP to determine how the robot should move to obtain more relevant samples to complete the map. IGDM leverages the strength of GW-GMRF, namely the ability to deal with wind currents and obstacles, and closes its control loop so that the robot’s exploration yields reliable gas estimates even in complex indoor environments, as demonstrated experimentally.

One relevant aspect of IGDM is that it shares the same approach as *infotaxis* [30], an information-driven search strategy, to locate the source of a gas distribution. Instead of following a predictable pattern or moving to locations with a higher gas concentration, both strategies (IGDM and *infotaxis*) focus on minimizing the uncertainty of the gas distribution, even if this means sampling at locations with no gas at all to confirm its absence [53]. Despite their differences, one being aimed at Gas Distribution Modeling (GDM) and the other at Gas Source Localization (GSL), it is reasonable to consider that they could be eventually brought together for mutual benefit. For instance, the robot could estimate the likely position of the gas source given an estimated gas map and then refine said estimate through CFD techniques once the source is confirmed. However, this warrants more research before it can be put into practice.

In this regard, one of our future goals is to develop a new GSL strategy similar to IGDM that also produces a probability map of gas source locations [7]. This could be especially relevant for safety-related applications such as gas leak detection, where the algorithm could provide a gas map to take immediate palliative actions (e.g., evacuation of human personnel) as well as information about the origin of the leak to plan for corrective actions (e.g., dispatching a robot that is equipped with a sealant gun). Still, before we can achieve this, we first have to further reduce the computational requirements of IGDMs current formulation. One option is changing the underlying grid map of GW-GMRF to one with a variable resolution. In this way, adjacent areas that share similar gas values could be bundled together and computed as a single unit. However, doing so implies that the complete lattice may change between iterations, which brings a whole new set of challenges. Another option might be to reduce the number of action combinations that are evaluated during each forward simulation. As discussed previously, the most immediate solution would be to replace the decision stage in the POMDP with a Monte Carlo Tree Search (MCTS) or a variation of Rapidly Exploring Random Trees (RRT), which would also help to mitigate the exponential cost of increasing the finite horizon beyond the values we have used in this work.

Lastly, we are also planning to extend the IGDM algorithm to multi-robot systems. Although our current implementation might work if run on several robots that explore the same environment, they would not be aware of each other’s intentions. Possibly, the best option is to include an additional term to the reward function that accounts for the relative position of the robots, as this would fit well under the current formulation. However, this is still a hypothetical approach, and more research is needed on this front. 

## Figures and Tables

**Table 1:** Selected values for the user-configurable parameters.

**GW-GMRF**		
**Parameter**	**Value**	**Units**
$\sigma_{gz}^{2}$	0.1	ppm
$\sigma_{gr}^{2}$	1.128	ppm
$\sigma_{d}^{2}$	$10^{8}$	ppm
$\sigma_{\zeta_{gz}}^{2}$	$1.44\times 10^{-4}$	$\mathrm{ppm}/s^{\frac{1}{2}}$
$\sigma_{wz}^{2}$	0.1	m/s
$\sigma_{wr}^{2}$	0.825	m/s
$\sigma_{wc}^{2}$	0.048	m/s
$\sigma_{wo}^{2}$	0.22	m/s
$\sigma_{gw}^{2}$	0.12	ppm·m/s
$\sigma_{\zeta_{wz}}^{2}$	$1.44\times 10^{-4}$	$m/s^{\frac{2}{3}}$
**POMDP**		
**Parameter**	**Value**	**Units**
coarse cell size	1.0	m
fine cell size	0.1	m
*H*	3	−
$d_{A}$ step size	1	m
γ	0.5	-
$\epsilon_{g}$	10	-
$k_{g}$	0.25	${\mathrm{ppm}}^{\frac{1}{2}}$
$k_{d}$	1	$m^{\frac{1}{2}}$
$k_{\theta }$	1/π	${\mathrm{rad}}^{\frac{1}{2}}$

## Data Availability

No new data were created or analyzed in this study. Data sharing is not applicable to this article.
